# Breaking the Stiffness: Functional and Radiological Results of Three Fixation Approaches in First MTP Arthrodesis

**DOI:** 10.3390/jcm14196923

**Published:** 2025-09-30

**Authors:** Serkan Aydin, Onder Ersan

**Affiliations:** 1 Department of Orthopedics and Traumatology, Ankara Etlik City Hospital, Ankara 06110, Turkey; 2 Department of Orthopedics and Traumatology, Ankara Diskapi Yildirim Beyazit Education and Research Hospital, Ankara 06110, Turkey; onersan@gmail.com

**Keywords:** hallux rigidus, first metatarsophalangeal joint, arthrodesis, fixation techniques, dorsal locking plate, crossed cortical screw, functional outcomes, foot surgery

## Abstract

**Objectives**: This study aimed to compare the clinical, functional, and radiological outcomes of three different fixation techniques—dorsal locking plate, crossed cortical screw, and a combination of both—used in first metatarsophalangeal (MTP) joint arthrodesis for advanced-stage hallux rigidus. The goal was to provide evidence-based guidance for surgical technique selection. **Methods**: This retrospective cohort study included 52 patients with advanced hallux rigidus (stage III–IV, Coughlin–Shurnas classification) who underwent surgical treatment between 2023 and 2025 at the Department of Orthopedics and Traumatology of Ankara Etlik City Hospital, with a minimum follow-up of one year. Patients were categorized into three groups according to the fixation technique used. Visual Analog Scale (VAS), American Orthopaedic Foot & Ankle Society (AOFAS) score, and Foot Function Index (FFI) were assessed using validated Turkish-language versions of the questionnaires. Radiological parameters included hallux valgus angle, first toe dorsiflexion angle, distal interphalangeal (DIP) arthritis, and radiographic union—defined as trabecular bridging across at least three cortices on weight-bearing anteroposterior and lateral radiographs. ANCOVA was performed with age as a covariate. **Results**: A total of 52 patients were included: Group 1 (dorsal plate fixation, n = 19), Group 2 (crossed cortical screw fixation, n = 16), and Group 3 (combined fixation, n = 17). Group 1 patients were significantly older (mean age: 64 ± 6 vs. 55 ± 6 and 59 ± 5 years; *p* < 0.001). After age adjustment, VAS pain scores were significantly higher in Group 1 compared to Group 3 (mean VAS: 2.8 ± 0.6 vs. 1.9 ± 0.5; *p* = 0.010). AOFAS scores did not differ significantly (*p* = 0.166), although Group 2 showed the highest median value (90 [70–93]). FFI scores differed significantly (*p* < 0.001), with Group 1 reporting worse outcomes (19 [17–31]) than Group 2 (15 [13–22], *p* = 0.03) and Group 3 (15 [11–16], *p* = 0.01). Dorsiflexion angle was significantly lower in Group 2 than Group 1 (median 19° vs. 27°; *p* = 0.04), though all remained within the physiological range. Radiographic union was achieved in 50/52 patients (96.2%), without significant intergroup differences (*p* = 0.612). Complications included two cases of wound dehiscence in Group 1; no infections, symptomatic non-union, malalignment, or hardware irritation were observed. **Conclusions**: Crossed cortical screw fixation yielded the most favorable functional outcomes, whereas the combined technique achieved the lowest postoperative pain scores. Dorsal plate fixation alone consistently underperformed. While outcomes were adjusted for age, residual confounding cannot be excluded. These results highlight the importance of tailoring fixation strategy to patient profile, with crossed screw and combined methods representing reliable choices for optimizing postoperative outcomes in advanced hallux rigidus.

## 1. Introduction

Hallux rigidus is a progressive degenerative arthropathy primarily affecting the first metatarsophalangeal (MTP) joint. It is characterized by chronic and worsening joint stiffness, pain particularly exacerbated during dorsiflexion, and the eventual development of dorsal osteophyte formation, especially in advanced stages of the disease [1]. As the condition progresses, cartilage degradation and osteoarthritic changes lead to mechanical blockages that severely impair gait and quality of life. The most frequently reported symptom among patients is painful restriction of dorsiflexion, commonly accompanied by the formation of a dorsal pseudo-exostosis, which further impairs toe mobility and causes footwear-related discomfort [2,3,4,5].

Treatment strategies for hallux rigidus vary depending on the severity of joint degeneration, functional limitations, and patient expectations. In early or moderate stages, conservative modalities such as activity modification, orthotic use, intra-articular injections, and nonsteroidal anti-inflammatory drugs (NSAIDs) may provide symptomatic relief. However, in advanced stages, these measures are often insufficient, and surgical intervention becomes necessary.

Several surgical techniques have been proposed for hallux rigidus, including soft tissue procedures (such as cheilectomy or tendon balancing), proximal phalangeal or metatarsal osteotomies, joint replacement (arthroplasty), and arthrodesis of the first MTP joint. Among these options, first MTP joint arthrodesis is widely regarded as the gold standard, particularly in cases with extensive cartilage loss and joint incongruity [6,7,8]. Arthrodesis reliably relieves pain, corrects deformity, and provides durable outcomes in terms of patient satisfaction and function.

While each technique has been shown to achieve satisfactory union rates, direct comparative data remain limited, particularly regarding functional outcomes, pain control, and complication profiles. Recent studies suggest that crossed screw fixation may have certain advantages over dorsal plating, including lower rates of implant-related irritation, reduced surgical exposure, and more favorable short-term outcomes in pain and function [9,10]. However, comparative evidence encompassing all three methods—especially using standardized outcome metrics—is scarce.

In light of these considerations, we hypothesize that crossed cortical screw fixation offers superior functional performance, as measured by American Orthopedic Foot and Ankle Society (AOFAS) scores and the Foot Function Index (FFI), and results in lower pain levels based on the Visual Analog Scale (VAS), when compared to both dorsal plate fixation and the combined technique. Therefore, the primary objective of this study is to systematically compare the one-year clinical, functional, and radiological outcomes of these three commonly used fixation techniques in patients undergoing first MTP joint arthrodesis for advanced hallux rigidus. By doing so, we aim to provide evidence-based guidance to assist surgeons in selecting the most appropriate fixation method tailored to patient-specific needs.

## 2. Materials and Methods

### 2.1. Study Design and Patient Selection

This study was designed as a retrospective cohort analysis. After receiving approval from the institutional ethics committee, we reviewed the records of patients treated for hallux rigidus between 20 January 2023, and 1 January 2025, at the Orthopedics and Traumatology Department of Ankara Etlik City Hospital. A total of 52 patients who underwent surgical treatment with documented follow-up of at least 12 months (mean follow-up: 12–18 months) were included in the analysis.

Patients were divided into three groups according to the type of fixation method used:

✓ Group 1: Dorsal locking plate fixation (n = 19)
✓ Group 2: Crossed cortical screw fixation (n = 16)
✓ Group 3: Combined dorsal locking plate and crossed cortical screw fixation (n = 17)

The choice of fixation method was determined by the treating surgeon based on intraoperative bone quality, deformity severity, and surgeon preference. Patients with osteoporotic bone or higher deformity load were more likely to receive plate or combined fixation, whereas crossed screws were generally preferred in younger patients with good bone stock. No formal randomization was performed due to the retrospective design of the study. In all cases, a dorsal surgical approach was used. Following joint preparation suitable for arthrodesis, fixation was performed according to the assigned method. All procedures were performed with the patient in the supine position under spinal or general anesthesia, with a pneumatic tourniquet applied to the thigh. A dorsal longitudinal incision was used in all cases. Joint surfaces were prepared by removing residual cartilage and subchondral drilling until bleeding cancellous bone was exposed. Fixation was performed according to group allocation: (i) dorsal locking plate with 3.5-mm titanium screws (Group 1), (ii) two crossed 3.0-mm cortical screws (Group 2), or (iii) combined fixation with both a dorsal plate and crossed screws (Group 3). Postoperatively, pain control was achieved using a standardized multimodal analgesia protocol consisting of intravenous paracetamol, nonsteroidal anti-inflammatory drugs (NSAIDs), and, if required, patient-controlled opioid analgesia during the first 24 h. There was no difference in analgesic regimen among the groups. All patients were immobilized in a below-knee walking boot for six weeks. Non-weight-bearing was advised for the first two weeks, followed by partial weight-bearing with crutches until week six, and progression to full weight-bearing thereafter. Rehabilitation protocol was uniform across all groups.

Inclusion criteria were as follows: age ≥ 18 years, clinical evidence of restricted range of motion at the first MTP joint, and radiographic findings consistent with MTP osteoarthritis. All patients were diagnosed with advanced hallux rigidus (corresponding to stage III–IV disease according to the Coughlin–Shurnas classification) [11]. Although formal staging was not consistently documented in the retrospective records, only patients with advanced disease were included, ensuring a relatively homogeneous cohort.

Exclusion criteria included: presence of peripheral neuropathy in the same limb, history of previous MTP joint surgery, inflammatory arthritis, interphalangeal joint arthritis, or prior foot/ankle arthroplasty that could affect functional outcomes.

The following data were recorded: demographic variables (age, sex), laterality (right/left), complication occurrence, and presence of distal interphalangeal (DIP) arthritis. Clinical assessments included:

✓ Visual Analog Scale (VAS) for pain
✓ American Orthopedic Foot and Ankle Society (AOFAS) score
✓ Foot Function Index (FFI)

**Figure 1:**
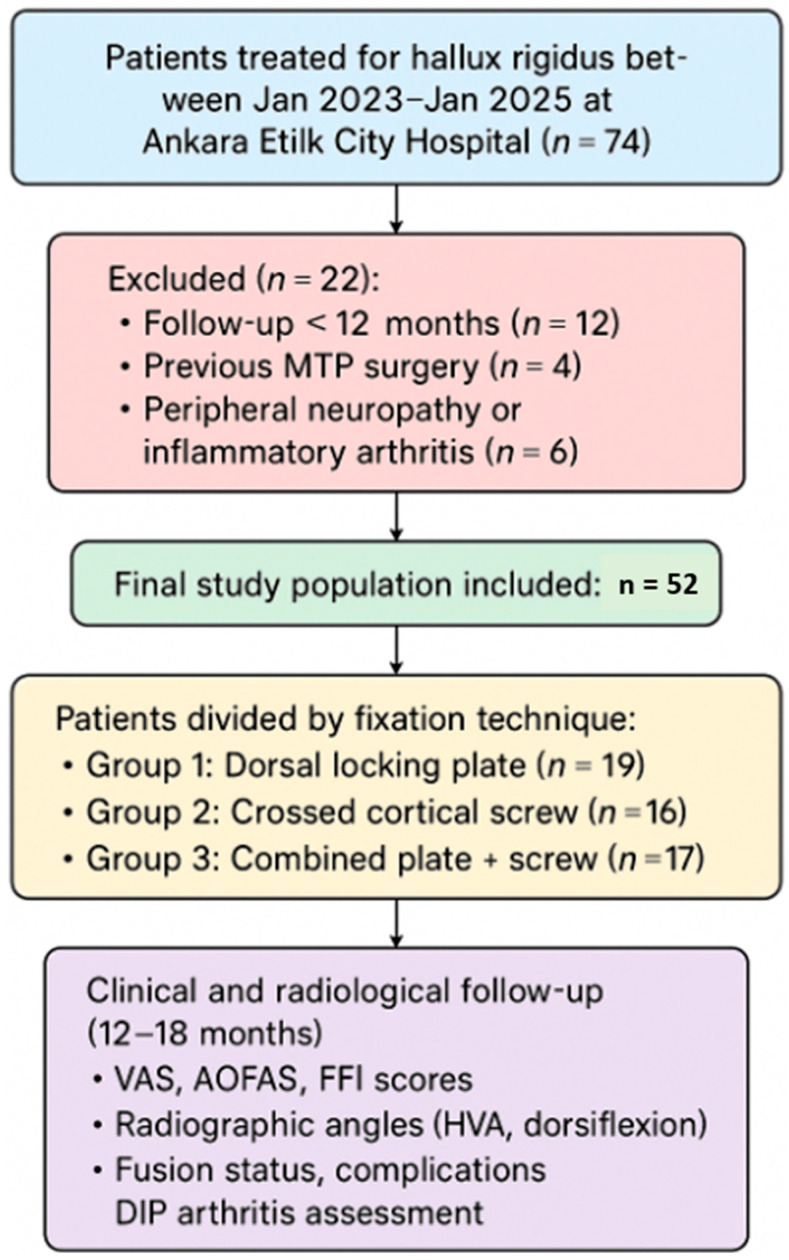
Flowchart of the study design.

The American Orthopedic Foot and Ankle Society (AOFAS) score and Foot Function Index (FFI) were collected using validated Turkish-language versions of the questionnaires, administered in an interview format by a trained orthopedic resident at follow-up visits [12]. Postoperative radiographic evaluations were conducted using weight-bearing anteroposterior and lateral foot X-rays at the last follow-up visit. Two independent observers assessed the presence of DIP arthritis. Hallux valgus angle (HVA) and first toe dorsiflexion angle were manually measured and recorded. Fusion status and wound-related complications were also evaluated. Radiographic union was defined as the presence of trabecular bridging across at least three cortices on weight-bearing anteroposterior and lateral radiographs, and union status was assessed at the final follow-up visit by two independent observers. All parameters were compared among the three fixation groups (Figure 1).

### 2.2. Statistical Analysis

All statistical analyses were conducted using SPSS Statistics version 20.0 (IBM Corp., Armonk, NY, USA). The normality of continuous variables was assessed using the Shapiro–Wilk test. Normally distributed data were expressed as mean ± standard deviation (SD), while non-normally distributed data were presented as median and interquartile range (IQR). Categorical variables were reported as frequencies and percentages. For comparisons of normally distributed continuous variables among groups, the Welch ANOVA test was employed. When significant differences were detected, the Games–Howell post hoc test was used. For non-normally distributed continuous variables, the Kruskal–Wallis test was applied, followed by Dunn–Bonferroni post hoc tests when appropriate. Comparisons of categorical variables were performed using the chi-square test or Fisher’s exact test, depending on expected cell counts. To evaluate the effect of fixation type on clinical outcomes while controlling for age as a potential confounding factor, analysis of covariance (ANCOVA) was conducted. Although Levene’s test indicated a violation of the assumption of homogeneity of variances for some outcome variables, results were interpreted with caution, and Bonferroni-adjusted pairwise comparisons were performed when a significant main effect was found. All outcomes were reported as age-adjusted means and standard errors (SE). Additionally, significant differences between fixation methods were visualized using network diagrams, where nodes represented fixation types and edges indicated statistically significant relationships. A *p*-value of <0.05 was considered statistically significant. Effect sizes were reported using partial eta-squared (η^2^) values.

## 3. Results

A total of 52 patients were included in the study, distributed across three fixation groups. Group 1 patients were significantly older than those in Groups 2 and 3 (*p* < 0.001). No significant differences were observed among the groups in terms of gender distribution (*p* = 0.360) or complication rates (*p* = 0.164). Complications included two cases of wound dehiscence requiring local wound care in Group 1. No infections, symptomatic non-union, malalignment, or hardware irritation were observed in any group during the follow-up period. Laterality showed a statistically significant difference (*p* < 0.001), with all Group 2 procedures performed on the left foot. The presence of distal interphalangeal (DIP) arthritis was more frequent in Group 1, although this did not reach statistical significance (*p* = 0.080) (Table 1).

No statistically significant difference was found among the three groups in terms of VAS (*p* = 0.06) and AOFAS scores (*p* = 0.09), although Group 2 demonstrated the highest median AOFAS score. However, FFI scores differed significantly among groups (*p* < 0.005), with Group 2 and Group 3 showing more favorable functional outcomes compared to Group 1. In terms of first phalanx dorsiflexion, Group 2 showed significantly lower angles compared to Group 1 (*p* < 0.05), although all values remained within the accepted normal range (15.9–26.9°). There was no significant difference in hallux valgus angle between groups (*p* = 0.949) (Table 2). Radiographic union was achieved in 50 of 52 patients (96.2%) overall, with no significant difference among fixation groups (Group 1: 18/19, 94.7%; Group 2: 16/16, 100%; Group 3: 16/17, 94.1%; *p* = 0.612). All cases that failed to demonstrate complete radiographic union were asymptomatic and did not require revision surgery.

**Figure 2:**
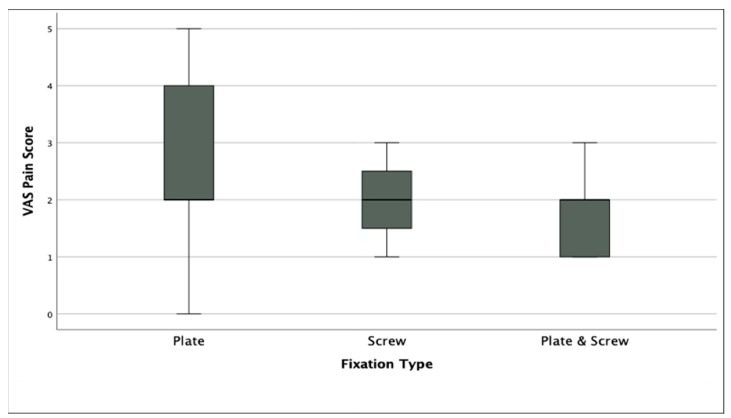
Boxplot showing Visual Analog Scale (VAS) pain scores among the three fixation groups.

Although the median VAS score was lower in the combined fixation and screw-only groups, the difference between groups did not reach statistical significance (*p* = 0.06) (Figure 2).

**Figure 3:**
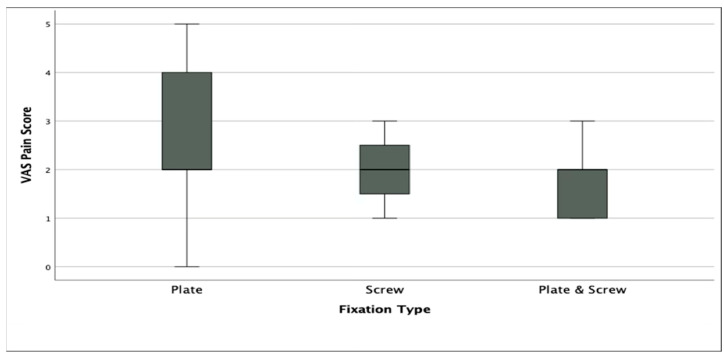
Boxplot depicting American Orthopedic Foot and Ankle Society (AOFAS) scores among the three fixation groups.

Although Group 2 (crossed screw fixation) had the highest median AOFAS score, the effect size was small and the difference did not reach statistical significance (*p* = 0.09) (Figure 3).

**Figure 4:**
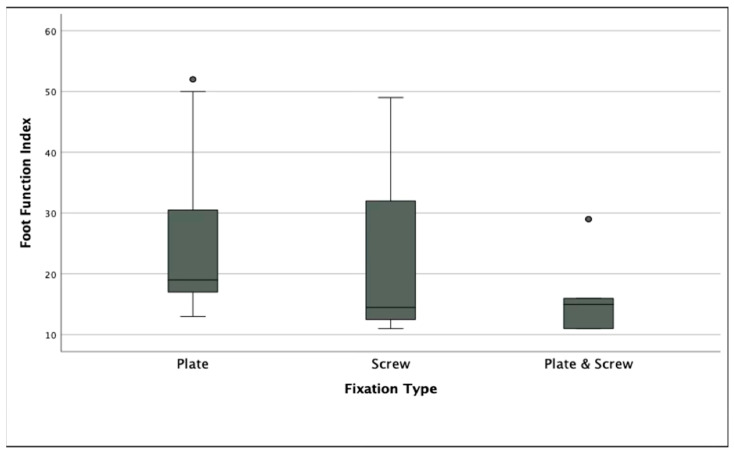
Boxplot displaying Foot Function Index (FFI) scores among the three fixation groups. Dots above the whiskers represent statistical outliers (values outside 1.5× IQR).

The highest FFI score was observed in Group 1, indicating poorer function. The difference among groups was statistically significant (*p* < 0.005) (Figure 3). Post hoc comparisons revealed that Group 1 had significantly worse FFI outcomes compared to both Group 2 (*p* = 0.03) and Group 3 (*p* = 0.01) (Figure 4).

**Figure 5:**
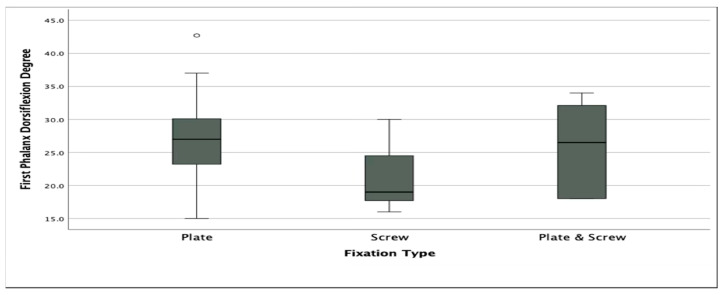
Boxplot illustrating first phalanx dorsiflexion angles among the three fixation groups. Circle above the whiskers represent statistical outliers (values outside 1.5× IQR).

While dorsiflexion angles in Group 2 remained within the accepted normal range (15.9–26.9°), the values were significantly lower compared to Group 1, where angles exceeded the average upper limit. This difference was statistically significant (*p* = 0.04) (Figure 5).

Analysis of covariance (ANCOVA) revealed a statistically significant effect of fixation type on VAS pain scores (*p* = 0.010) after adjusting for age. While initial unadjusted comparisons showed no significant differences, the adjusted analysis indicated that Group 1 had significantly higher VAS scores than Group 3. For AOFAS scores, although fixation type was not a significant factor (*p* = 0.166), age was found to significantly influence outcomes (*p* = 0.010). FFI scores were significantly affected by both fixation type (*p* < 0.001) and age (*p* < 0.001), with the largest effect size among all variables (partial η^2^ = 0.621 for age). For first toe dorsiflexion, age was a significant covariate (*p* = 0.018), whereas fixation type did not show a statistically significant effect after adjustment (*p* = 0.231). Hallux valgus angle was not significantly affected by either fixation type or age (Table 3).

**Figure 6:**
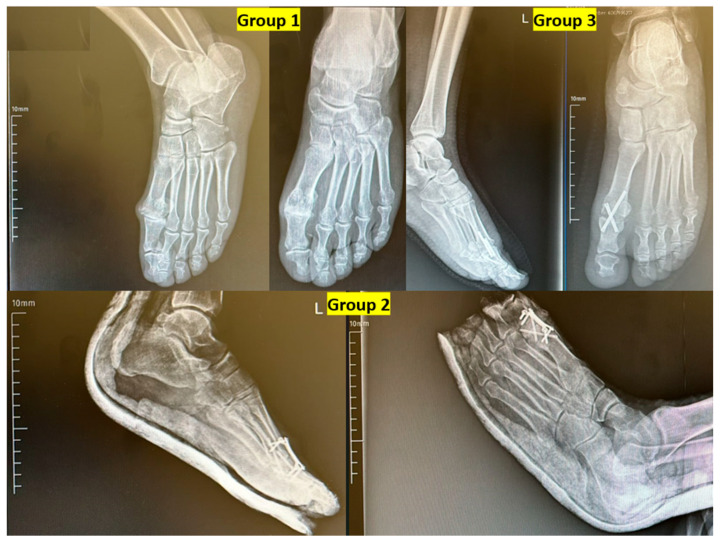
Representative postoperative radiographs of patients from each fixation group. Group 1: Dorsal locking plate fixation. Group 2: Crossed cortical screw fixation. Group 3: Combination of dorsal plate and crossed screws. L: Left.

While dorsiflexion angles in Group 2 remained within the accepted normal range (15.9–26.9°), the values were significantly lower compared to Group 1, where angles exceeded the average upper limit. This difference was statistically significant (*p* = 0.04) (Figure 6).

**Figure 7:**
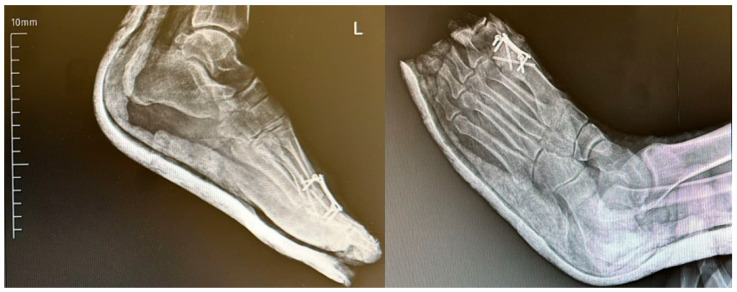
Lateral radiographic view demonstrating optimal dorsiflexion angle following first metatarsophalangeal (MTP) joint arthrodesis. L: Left.

A lateral radiographic view demonstrating the arthrodesis angle is shown in Figure 6. The image illustrates proper alignment of the first toe with approximately 10–15° of dorsiflexion, which is considered the ideal postoperative fusion position for restoring gait mechanics and ensuring optimal functional outcomes (Figure 7).

## 4. Discussion

In this study, we compared the clinical and functional outcomes of three different fixation techniques used for first metatarsophalangeal (MTP) joint arthrodesis in the treatment of hallux rigidus over a one-year follow-up period across three groups: Group 1—dorsal locking plate fixation; Group 2—crossed cortical screw fixation; Group 3—a combination of both methods.

In the literature, surgical treatment options for advanced hallux rigidus often focus on either first MTP joint arthrodesis or arthroplasty. Several studies comparing these approaches using AOFAS, VAS, and MOXFQ scores have concluded that arthrodesis generally provides superior outcomes [13,14].

In a study by Dong Than Minh et al., 58 patients with hallux rigidus were treated with either dorsal locking plate or crossed cortical screw fixation. The authors reported comparable 6-month clinical and radiological outcomes between the two groups; however, patients in the screw group experienced fewer implant-related complaints [10]. Similarly, a study by Verinder S. Sidhu et al. involving 108 patients compared the same two techniques and found no significant differences in fusion rates or complication profiles [9].

To our knowledge, no previous study has conducted a direct comparison of all three fixation techniques evaluated in our study. Our findings, based on VAS, AOFAS, and FFI scores, contribute to the limited body of literature on this topic. Although the initial comparison did not show a significant difference in pain scores (VAS), the ANCOVA analysis—adjusted for age—revealed that Group 1 had significantly higher pain levels compared to Group 3. This highlights the potential influence of age on subjective pain reporting and suggests that dorsal plate fixation may be associated with greater postoperative discomfort, particularly in older patients.

Although Group 2 showed the highest AOFAS scores in both age-adjusted and unadjusted analyses, the differences were not statistically significant, and the effect size was small, indicating limited clinical relevance. In contrast, Foot Function Index (FFI) scores were significantly worse in Group 1, both before and after adjustment for age, suggesting inferior functional outcomes with dorsal plate fixation alone. In line with previous reports demonstrating high union rates following first MTP arthrodesis, our cohort achieved radiographic fusion in more than 95% of cases, with no significant intergroup difference. This reinforces the reliability of all three fixation techniques in achieving union, while functional outcomes distinguished their performance profiles.

Given that dorsal locking plates are frequently used as the default technique for first MTP joint arthrodesis, our findings warrant caution. The inferior FFI outcomes observed in Group 1 suggest that crossed cortical screw fixation—or its combination with a dorsal plate—may offer better functional recovery and should be considered as viable alternatives, particularly in younger and more active patients.

In the present study, the normal range for first toe dorsiflexion was considered to be approximately 15.9–26.9 degrees [15]. While dorsiflexion angles in Group 2 remained within this physiological range, the values were significantly lower than those in Group 1, where the angles tended to exceed the upper limit of the normal range. This difference was found to be statistically significant. Although the difference in dorsiflexion angle between groups reached statistical significance, all values remained within the accepted physiological range (15–30°). Thus, while statistically notable, this difference is unlikely to have meaningful clinical implications in terms of gait mechanics or functional recovery. This highlights the importance of distinguishing statistical significance from clinical relevance in interpreting our results.

In a retrospective analysis conducted by Yang S. Kang et al., three fixation techniques were compared: dorsal locking plate, crossed cortical screw, and staple fixation [16]. Although the staple method yielded the highest fusion rates, the authors reported that dorsal plate fixation provided superior outcomes in terms of lower complication rates, improved mechanical stability, earlier mobilization, and better functional recovery. However, the combined dorsal plate plus crossed screw fixation technique evaluated in our study was not included in their analysis. Our results suggest that dorsal plate fixation alone may be inferior to other fixation strategies, particularly in terms of functional outcomes.

Supporting this notion, a retrospective cohort study by Amir R. Kachooei et al., which included 372 patients and 378 first MTP arthrodesis procedures, demonstrated that dorsal locking plate fixation was associated with significantly higher rates of symptomatic non-union and reoperation compared to crossed cortical screw fixation [17]. These findings are in alignment with the outcomes of our study and further validate the concern regarding the use of dorsal plate fixation as a standalone technique.

Based on the findings of this study, it can be concluded that the choice of surgical technique for first MTP joint arthrodesis should be guided primarily by functional outcomes, while also considering clinical, radiological, and demographic factors. Our analysis revealed that VAS pain scores were significantly influenced by age. After adjusting for age, patients in Group 1 exhibited higher pain levels compared to those in other groups. This suggests that, in the absence of age differences, patients undergoing dorsal plate fixation might have required greater postoperative analgesia.

Regarding complications during the first postoperative year, wound-related problems were observed in two patients in Group 1, one of whom required implant removal due to persistent drainage. In the study by Maj Patrick D. Grimm et al., the most common complications following first MTP joint arthrodesis included non-union, interphalangeal arthritis, and sesamoid pain [18]. These findings were echoed by Thomas S. Roukis et al., who similarly identified non-union as the predominant complication in their series [19,20].

In a retrospective study involving 178 cases, Weigelt et al. compared two fixation methods: 97 patients were treated with dorsal locking plates supported by interfragmentary screws, and 81 patients received independent interfragmentary compression screws without plates. The overall non-union rate was reported as 6.2% (11 out of 178 cases) [21]. In contrast, no statistically significant differences were identified in our study with respect to non-union, interphalangeal arthritis, or sesamoid pain between fixation groups. However, it should be noted that our findings are limited to a 12-month follow-up period. Longer-term follow-up may provide more comprehensive insights into the durability and safety profiles of these fixation techniques.

Previous studies have suggested that the optimal arthrodesis position for the first MTP joint includes approximately 10–20° of valgus and 10–15° of dorsiflexion [22,23]. However, a study by Van Doeselaar et al. reported no clear association between fusion angles and functional outcomes [24]. In contrast, our findings indicate that Group 2 achieved fusion angles closest to the recommended alignment. When these radiographic findings are considered alongside the superior functional scores observed in this group, it suggests that proper arthrodesis positioning may positively influence postoperative functional outcomes.

### Limitations of the Study

This study has some limitations. First, its retrospective design inherently introduces the potential for selection bias. Notably, the mean age of patients in Group 1 was significantly higher than that in the other groups. To minimize the influence of this confounding factor, we applied analysis of covariance (ANCOVA) to statistically adjust for age differences. Second, the study was conducted at a single center with a relatively small sample size and a limited follow-up period of one year. Third, although validated Turkish versions of the AOFAS and FFI questionnaires were used, data were obtained through interviewer-administered assessments, which may introduce reporting bias. Another limitation is the lack of detailed information on potential confounding variables such as BMI, smoking status, diabetes, and peripheral vascular disease, which are known to influence bone healing and functional recovery. Although we performed age adjustment, residual confounding cannot be fully excluded. ASA scores were not consistently documented in retrospective records, representing a limitation. These factors restrict the generalizability of the findings. Therefore, future prospective, multicenter, and randomized controlled trials with larger cohorts and longer follow-up durations are warranted to validate our results.

## 5. Conclusions

In conclusion, our findings suggest that the choice of fixation technique in first MTP joint arthrodesis carries distinct clinical implications. Crossed cortical screws provided the most favorable functional outcomes, whereas the combined fixation technique was associated with the lowest postoperative pain scores after age adjustment. Dorsal plate fixation alone consistently yielded inferior results in both domains. Although complication rates were comparable, the potential influence of age on outcome measures must be acknowledged despite statistical adjustment. These results indicate that both crossed screw and combined fixation methods are reliable strategies, and the final choice should be individualized according to patient profile—balancing functional recovery, pain control, and demographic characteristics.

## Figures and Tables

**Table 1:** Comparison of Demographic Characteristics Among Fixation Groups.

Variables	Total (n = 52)	Fixation Type			*p*-Value
		Group 1 (n = 19)	Group 2 (n = 16)	Group 3 (n = 17)	
Age * (years)	60 ± 7	64 ± 6	55 ± 6	59 ± 5	<0.001
Gender (Female) †	36 (69.2%)	12 (63.2%)	10 (62.5%)	14 (82.4%)	0.360
Laterality (Left) †	22 (42.3%)	8 (52.1%)	18 (100%)	4 (23.5%)	<0.001
Complications †	2 (3.8%)	2 (10.5%)	0 (0%)	0 (0%)	0.164
Distal Interphalangeal Arthritis †	9 (17.3%)	5 (26.3%)	0 (0%)	4 (23.5%)	0.080

*: Data are presented as mean ± standard deviation. †: Data are presented as number of patients (percentage).

**Table 2:** Comparison of Functional, Clinical, and Radiological Outcomes Between Fixation Types.

Variables	Total (n = 52)	Fixation Type			*p*-Value
		Group 1 (n = 19)	Group 2 (n = 16)	Group 3 (n = 17)	
VAS ‡	2 (1–3)	2 (2–4)	2 (2–3)	2 (1–2)	0.06
AOFAS ‡	86 (77–91)	83 (70–90)	90 (70–93)	85 (83–94)	0.09
Foot Function Index ‡	15 (14–29)	19 (17–31)	15 (13–22)	15 (11–16)	<0.005
First Phalanx Dorsiflexion ‡	25.8 (18–30)	27 (22.8–30.2)	19 (17.7–24.5)	26.5 (18–32.1)	<0.05
Hallux Valgus Angle ‡	15.9 (9.8–20.8)	13.9 (9.7–22)	14.9 (7.8–22)	15.9 (9.9–17)	0.949

‡: Values are presented as median (25th–75th percentile).

**Table 3:** ANCOVA Results for Clinical and Radiological Outcomes Adjusted for Age.

Outcome Variables	df	F	*p*-Value	Partial Eta^2^	R^2^ (Adjusted)
VAS	Fixation Type (2)	5.109	0.010	0.176	0.176 (0.124)
	Age (1)	2.361	0.131	0.047	
AOFAS	Fixation Type (2)	1.866	0.166	0.072	0.192 (0.142)
	Age (1)	7.118	0.010	0.129	
Foot Function Index	Fixation Type (2)	10.853	<0.001	0.311	0.661 (0.640)
	Age (1)	78.620	<0.001	0.621	
First Phalanx Dorsiflexion	Fixation Type (2)	1.512	0.231	0.059	0.248 (0.202)
	Age (1)	6.037	0.018	0.112	
Hallux Valgus Angle	Fixation Type (2)	0.100	0.905	0.004	0.019 (−0.042)
	Age (1)	0.867	0.356	0.018	

## Data Availability

The original contributions presented in the study are included in the article, further inquiries can be directed to the corresponding author.

## References

[1] Lim B., Jassim S., Kilkenny C., Lyons F., Shaalan M. (2025). Crossed screws versus plating supplemented with an interfragmentary screw in first metatarsophalangeal joint fusion: A systematic review and meta-analysis. J. Foot Ankle Surg..

[2] de Bot R., Veldman H.D., Eurlings R., Stevens J., Hermus J.P.S., Witlox A.M. (2022). Metallic hemiarthroplasty or arthrodesis of the first metatarsophalangeal joint as treatment for hallux rigidus: A systematic review and meta-analysis. Foot Ankle Surg..

[3] Massimi S., Caravelli S., Fuiano M., Pungetti C., Mosca M., Zaffagnini S. (2020). Management of high-grade hallux rigidus: A narrative review of the literature. Musculoskelet. Surg..

[4] Galois L., Hemmer J., Ray V., Sirveaux F. (2020). Surgical options for hallux rigidus: State of the art and review of the literature. Eur. J. Orthop. Surg. Traumatol..

[5] de Buys M., Saragas N.P., Ferrao P.N.F. (2025). Why I Want Bunion Surgery-the Patient’s Preoperative and Postoperative Perspective. Foot Ankle Int..

[6] Budde K., Claassen L., Plaass C., Stukenborg-Colsman C., Daniilidis K., Yao D. (2024). Synthetic cartilage implant vs. first metatarsophalangeal arthrodesis for the treatment of hallux rigidus. Arch Orthop. Trauma Surg..

[7] Colo G., Fusini F., Alessio-Mazzola M., Samaila E.M., Formica M., Magnan B. (2022). Interposition arthroplasty with bovine collagenous membrane for hallux rigidus: A long-term results retrospective study. Foot Ankle Surg..

[8] Mosca M., Caravelli S., Vocale E., Fuiano M., Massimi S., Di Ponte M., Censoni D., Grassi A., Ceccarelli F., Zaffagnini S. (2022). Hallux valgus associated to osteoarthritis: Clinical-radiological outcomes of modified SERI technique at mid- to long-term follow-up. A retrospective analysis. Foot Ankle Surg..

[9] Sidhu V.S., Kim C.J., Pedowitz D.I., Pagliaro A.J., Fuchs D., O’Neil J.T., Daniel J., Shakked R., Tsai J., Winters B.S. (2023). Screw Only Fixation vs Plate Fixation For 1st MTPJ Arthrodesis: A Comparative Retrospective Study. Foot Ankle Orthop..

[10] Tran-Minh D., Poirot-Seynaeve B., Vialla T., Ohl X., Diallo S., Siboni R. (2024). Comparison of the outcomes of first metatarsophalangeal joint arthrodesis by locking plate and compression screw in patients with severe hallux valgus or hallux valgus revision. Orthop. Traumatol. Surg. Res..

[11] Coughlin M.J., Shurnas P.S. (2003). Hallux rigidus. Grading and long-term results of operative treatment. J. Bone Joint Surg. Am..

[12] Ziroglu N., Birinci T., Koluman A., Sahbaz Y., Ciftci M.U., Baca E., Duramaz A. (2023). Reliability and Validity of the Turkish Version of the American Orthopaedic Foot and Ankle Society Hallux Metatarsophalangeal-Interphalangeal Joint Scale. Foot Ankle Spec..

[13] Fieschi S., Saffarini M., Manzi L., Fieschi A. (2017). Mid-term outcomes of first metatarsophalangeal arthroplasty using the Primus FGT double-stemmed silicone implants. Foot Ankle Surg..

[14] Sanchez Guzman J., Gallo Oropeza R., Reyes Donado M., Martin Oliva X., Diaz Sanchez T. (2024). Arthrodesis vs arthroplasty for moderate and severe Hallux rigidus: Systematic review. Foot Ankle Surg..

[15] Fragniere N., Kameni-Hekam M., Cisse A., Vienne P. (2024). Primary Isolated Arthrodesis of the First Metatarsophalangeal Joint for Hallux Rigidus: Clinical, Radiologic, and Pedobarographic Evaluation. Foot Ankle Orthop..

[16] Kang Y.S., Bridgen A. (2022). First metatarsophalangeal joint arthrodesis/fusion: A systematic review of modern fixation techniques. J. Foot Ankle Res..

[17] Kachooei A.R., Moncman T., Ghayyad K., Raikin S., Daniel J. (2025). Plate Fixation and Hallux Valgus Deformity Impact on Nonunion Rates in First Metatarsophalangeal Arthrodesis A Retrospective Cohort Study. Foot Ankle Spec..

[18] Grimm M.P.D., Irwin T.A. (2022). Complications of Hallux Rigidus Surgery. Foot Ankle Clin..

[19] Roukis T.S., Townley C.O. (2003). BIOPRO resurfacing endoprosthesis versus periarticular osteotomy for hallux rigidus: Short-term follow-up and analysis. J. Foot Ankle Surg..

[20] Roukis T.S. (2011). Nonunion after arthrodesis of the first metatarsal-phalangeal joint: A systematic review. J. Foot Ankle Surg..

[21] Weigelt L., Redfern J., Heyes G.J., Butcher C., Molloy A., Mason L. (2021). Risk Factors for Nonunion After First Metatarsophalangeal Joint Arthrodesis With a Dorsal Locking Plate and Compression Screw Construct: Correction of Hallux Valgus Is Key. J. Foot Ankle Surg..

[22] Aas M., Johnsen T.M., Finsen V. (2008). Arthrodesis of the first metatarsophalangeal joint for hallux rigidus--optimal position of fusion. Foot.

[23] Huang D., Png W., Rikhraj I.S., Cher E.W.L. (2025). Cheilectomy, Osteotomy, Microfracture, and Matrix-Induced Chondrogenesis (COMM): A Novel Combined Procedure for Treating Hallux Rigidus. Cartilage.

[24] van Doeselaar D.J., Heesterbeek P.J., Louwerens J.W., Swierstra B.A. (2010). Foot function after fusion of the first metatarsophalangeal joint. Foot Ankle Int..

